# Supplementary material for: Visual Function and Neuropsychological Profiling of Idiopathic Infantile Nystagmus
Source: Brain Sci. 2023 Sep 20;13(9):1348. doi: 10.3390/brainsci13091348 (PMC10526276; doi:10.3390/brainsci13091348)
Supplement: Supplementary file 1 [file brainsci-13-01348-s001.zip › brainsci-2578886-supplementary.pdf]

**Supplementary Table S1**. Clinical and diagnostic evaluations. ERG: electroretinogram. VEP: visual evoked potential. OCT: optical coherence tomography. MRI: magnetic resonance imaging.

| Diagnostic exams | N=60 | | Results | n | % of performed |
|---|---|---|---|---|---|
| ERG | 47 (80%) | | Normal | 42 | 89 |
| | | | Not interpretable due to scarce collaboration | 5 | 11 |
| VEPs | 53 (88%) | | Normal | 15 | 28 |
| | | | Aspecific alterations | 38 | 72 |
| OCT | 0 | | / | / | / |
| Brain MRI | 24 (40%) | | Normal | 24 | 100 |
| Genetic testing | 23 (38%) | | Negative | 12 | 52 |
| | | | Ongoing | 5 | 22 |
| | | | FRMD7 | 6 | 26 |
| Ophthalmologic follow-up | 60 (100) | Anterior segment | Normal | 60 | 100 |
| | | Eye lens | Normal | 60 | 100 |
| | | Fundus oculi | Normal | 60 | 100 |
| Neuropediatric follow-up | 60 (100) | | Normal | 60 | 100 |

**Supplementary Table S2.** Cognitive test performed and results according to scoring rules of each test**.** A total of 27 patients (17 M, 10 F, mean age: 9.5 y, range: 4.3-17.8 y) underwent the cognitive assessment as explained in the methods section, according to their age. Wechsler preschool and primary scale of intelligence, fourth edition (WPPSI-IV); verbal comprehension index (VCI); visual–spatial index (VSI); fluid reasoning index (FRI); working memory index (WMI); processing speed index (PSI); and full-scale IQ (FSIQ). Wechsler preschool and primary scale of intelligence, third edition: verbal comprehension index (VCI); performance index (PI); processing speed index (PSI); and total intelligence quotient (TIQ). Wechsler intelligence scale for children (WISC-IV) and Wechsler adult intelligence scale (WAIS-IV): verbal comprehension index (VCI); perceptual reasoning index (PRI); working memory index (WMI); processing speed index (PSI); and intelligence quotient (IQ).

| IQ test (n=27) | Index | Performed n (%) | Not interpretable (%) | Index <85 (% of performed) | Index >/=85 (% of performed) |
|---|---|---|---|---|---|
| **WPPSI-IV (n=2)** | VCI | 2 (100) | 0 | 0 | 2 (100) |
| | VSI | 2 (100) | 0 | 0 | 2 (100) |
| | FRI | 2 (100) | 0 | 0 | 2 (100) |
| | WMI | 2 (100) | 0 | 0 | 2 (100) |
| | PSI | 2 (100) | 0 | 0 | 2 (100) |
| | FSIQ | 2 (100) | 0 | 0 | 2 (100) |
| **WPPSI-III (n=5)** | VCI | 5 (100) | 0 | 0 | 5 (100) |
| | PRI | 5 (100) | 0 | 0 | 5 (100) |
| | PSI | 4 (80) | 1 (20) | 0 | 4 (100) |
| | IQ | 5 (100) | 0 | 0 | 5 (100) |
| **WISC-IV (n=18)** | VCI | 18 (100)) | 0 | 0 | 18 (100) |
| | PRI | 18 (100) | 0 | 0 | 18 (100) |
| | WMI | 17 (94) | 1 (6) | 1 (6) | 16 (94) |
| | PSI | 18 (100) | 0 | 5 (28) | 13 (72) |
| | IQ | 15 (83) | 3 (17) | 1 (7) | 14 (93) |
| **WAIS-IV (n=2)** | VCI | 2(100) | 0 | 0 | 2 (100) |
| | PRI | 2 (100) | 0 | 0 | 2 (100) |
| | WMI | 2 (100) | 0 | 0 | 2 (100) |
| | PSI | 2 (100) | 0 | 0 | 2 (100) |
| | IQ | 2 (100) | 0 | 0 | 2 (100) |
